# The analysis of HPV integration sites based on nanopore sequencing and the profiling changes along the course of photodynamic therapy

**DOI:** 10.1186/s12885-023-11538-2

**Published:** 2023-11-02

**Authors:** Xiulan Li, Xiaoke Wei, Xin Liu, Nan Wang, Fuqiang Xu, Xingyu Liu, Yanmei Li, Yuxiang Zhou, Huadong Tang, Meina Bian, Ying Hou, Lili Zhang, Weiwei Wang, Qing Liu

**Affiliations:** 1 Department of Gynecology, Beijing Youan Hospital, Capital Medical University, Beijing, 100069 China; 2 Geneis, Bldg A, 5 Guangshun North Street, Beijing, 100102 China

**Keywords:** Human papillomavirus, Cervical carcinomas, Photodynamic therapy, Nanopore sequencing

## Abstract

**Objective:**

To detect the HPV genotype and integration sites in patients with high-risk HPV infection at different stages of photodynamic therapy using nanopore technology and to evaluate the treatment effect.

**Methods:**

Four patients with HPV infection were selected and subjected to photodynamic therapy, and cervical exfoliated cell was sampled at before treatment, after three courses of treatment and six courses of treatment, their viral abundance and insertion sites were analyzed by nanopore technology, and pathological examinations were performed before and after treatment. In this study, we developed a novel assay that combined viral sequence enrichment and Nanopore sequencing for identification of HPV genotype and integration sites at once. The assay has obvious advantages over qPCR or NGS-based methods, as it has better sensitivity after viral sequences enrichment and can generate long-reads (kb to Mb) for better detection rate of structure variations, moreover, fast turn-around time for real-time viral sequencing and analysis.

**Results:**

The pathological grade was reduced in all four patients after photodynamic therapy. Virus has been cleared in two cases after treatment, the virus amount reduced after treatment but not completely cleared in one case, and two type viruses were cleared and one type virus persisted after treatment in the last patient with multiple infection. Viral abundance and the number of integration sites were positively correlated. Gene enrichment analysis showed complete viral clearance in 1 patient and 3 patients required follow-up.

**Conclusion:**

Nanopore sequencing can effectively monitor the abundance of HPV viruses and integration sites to show the presence status of viruses, and combined with the results of gene enrichment analysis, the treatment effect can be dynamically assessed.

Cervical cancer is one of the most common female malignant tumor, and the incidence rate ranks first among malignant tumor of female reproductive system. According to the data of 2020 from World Health Organization (WHO), there were about 604 thousand new cases and 342 thousand deaths worldwide every year, which seriously threatened women's lives and health. Since professor Hausen of Germany found that Human papillomavirus (HPV) was closely related to the occurrence of cervical cancer in the late 1970s, HPV detection was gradually used for cervical cancer screening. HPVs are a heterogeneous group of nonenveloped, double-stranded, circular DNA viruses of 8000 nucleotides in size that infect the basal layer of the epithelium of skin (cutaneous HPV types) and mucous tissues (mucosal HPV types) [1, 2]. A phylogenetic tree construction based on the nucleotide sequence of the L1 gene classified the HPV types into five genera – alpha, beta, gamma, mu and nu [3, 4]. The Alpha papillomaviruses are further classified into high-risk (HR) types and low-risk (LR) types, according to their potential to cause anogenital cancer [1, 3, 5]. Persistent infection with HR-HPV genotypes triggers the integration of HPV DNA into the host genome that eventually leads to the accumulation of chromosomal damages and genome destabilisation in the infected cells [6–9]. The frequency of viral integration increases with the severity of cervical intraepithelial neoplasia (CIN) of stages I, II, III and the integrated form of the HPV DNA is found in the vast majority of cervical cancer cases [9, 10]. The WHO published the latest screening and treatment guidelines for cervical precancerous lesions in 2021 and recommend the use of HPV DNA testing as a primary screening for cervical cancer [11]. At present, polymerase chain reaction (PCR) technology is mainly used to detect HPV in clinic, but this method can not check the activity of HPV and the status of genome integration. According to the previous reports, the integration site of HPV is closely related to the occurrence, progression and recurrence of cancer. The main molecular mechanism may be the HPV insertion will affect the expression of upstream and downstream genes near the integration sites, the protein structure and the genomic structure’s instability [12, 13]. In recent years, the liquid microarray enrichment technology combined with next-generation sequencing technology can detect the subtypes of HPV, load, virus integration, insertion sites, etc. However, the read length is short, the accuracy of this method may be affected, which may lead to the low total detection rate of integration sites [9, 14]. Nanopore sequencing technology, as a new generation of sequencing technology in recent years, has the advantages of high throughput, ultra-long reading length, real-time sequencing, small and easy to carry instruments. It has obvious advantages for testing genome structure variation, virus insertion sites and insertion structure detection. It is widely used in many life sciences and clinical medical research.

Aminolevulinic acid photodynamic therapy (ALA-PDT) is a combination of medicine and machinery. After exogenous ALA is given, it is selectively absorbed by proliferating active cells. After a series of enzymatic reactions, a large number of photosensitive substances protoporphyrin IX is generated in mitochondria. After irradiation with a specific wavelength light source, reactive oxygen species such as singlet oxygen and oxygen free radicals are produced, which can selectively destroy the diseased tissue and achieve the purpose of treatment. In recent years, ALA-PDT has been used to treat cervical intraepithelial neoplasia (CIN). Studies have shown that the histological remission rate of ALA-PDT in the treatment of CIN I was 75%—86% in 3–6 months; after the treatment, the histological remission rate of CIN II reached 90% in 3–12 months [15]. Our research also achieved good results in the treatment of cervical intraepithelial neoplasia and cervical HPV infection with ALA-PDT [16]. In our study, nanopore sequencing technology was used to detect the HPV virus sequence of patients' samples before and after photodynamic therapy for cervical intraepithelial neoplasia. The proportion of HPV and the number of inserted human genome were counted and analyzed. Compare the HPV integration hotspots and related genes in samples at different stages before and after treatment of cervical lesions, and try to find the integration site spectrum of genes related to the progression and poor prognosis of cervical lesions. Then evaluate the feasibility of the detection methodology as an accurate companion diagnostic tool, and provide basic research data for prognosis evaluation and the adjustment of individual treatment plan.

## Study design and ethics

Four patients with cervical intraepithelial neoplasia complicated with high-risk HPV infection who were admitted to the Department of Gynecology, Beijing You'an Hospital Affiliated to Capital Medical University from January 2021 to December 2021 were selected as the research objects. Two cases’ histopathology of cervical biopsy were CIN I while the other two were CIN II. The cases aged 30–52 years (Table 1). All the cases completed two stages of treatment [3 times of photodynamic therapy (3 PDT) and 6 times of photodynamic therapy (6 PDT)]. All patients were aware of this study and signed informed consent. This study was approved by the Ethics Committee of Beijing You'an Hospital jing you ji lun zi [2019]003.

**Table 1:** Patient enrollment information

Sample ID	Age	Sample type	Therapy stage	HPV type(qPCR)	TCT	Postoperative pathological
B02	30	Cervical exfoliated cell	Before treatment	16	negative	CIN I
B02-1	30	Cervical exfoliated cell	3 PDT	negative	negative	N/A
B02-2	30	Cervical exfoliated cell	6 PDT	negative	negative	cervicitis
B15	33	Cervical exfoliated cell	Before treatment	52	ASCUS	CIN II
B15-1	33	Cervical exfoliated cell	3 PDT	negative	negative	N/A
B15-2	33	Cervical exfoliated cell	6 PDT	negative	negative	CIN I
B16	52	Cervical exfoliated cell	Before treatment	56、58、59	LSIL	CIN I
B16-1	52	Cervical exfoliated cell	3 PDT	56、59	negative	N/A
B16-2	52	Cervical exfoliated cell	6 PDT	56、59	negative	cervicitis
B19	30	Cervical exfoliated cell	Before treatment	52、58	LSIL	CIN II
B19-1	30	Cervical exfoliated cell	3 PDT	negative	negative	N/A
B19-2	30	Cervical exfoliated cell	6 PDT	negative	negative	cervicitis

### DNA extraction

The cervical exfoliated cell was collected with instructions by the disposable brush for cervix’s sampling kit (YIGUOREN biological technology, Hangzhou, China) and were put into 10 ml of preservation solution. Then 5 mL cervical exfoliated cells suspension was divided into a 1.5 mL centrifuge tube and centrifuged at 12,000–15000 rpm for 3 min. The supernatant was removed and the cell pellet was extracted and purified using TIANamp Micro DNA Kit (TIANGEN BIOTECH (BEIJING) DP316) according to the instructions. The extracted DNA was quantified using Qubit3.0, and the fragment length was inspected using Agarose gel electrophoresis. DNA samples with size > 5000 bp and more than 200 ng were further processed for library construction.

### Library construction

150 ng total DNA were sheared with ultrasound according to the optimized interruption conditions (M220 Focused-Ultrasonicator was used): Peak power: 25, Duty factor: 10, Cycles/burst: 500, Avg power: 2.5. The sheared DNA was further end-repaired and A-tailed using the NEBNext® Ultra IIEnd-prep reaction buffer and enzyme mix, and 1 × VAHTS® DNA Clean Beads (N411-02; Nanjing Vazyme Biotech Co Ltd. China) were used to remove short fragments to ensure library size larger than 500 bp, then PCR Barcoding Kit (SQK-PBK004; Oxford Nanopore Technologies, UK) was used for library construction according to instruction. The generated library was quantified with the Qubit dsDNA HS Assay Kit (Thermo Fisher Scientific, MA, United States) using the Qubit 3.0 fluorometer (Invitrogen, CA, United States), the size distribution of library was checked using the Agilent 2100 TapeStation (Agilent Technologies, CA, United States). The typical size range of the library was from 0.6 to 9Kbp.

### HPV sequences enrichment and nanopore sequencing

The probe set of HPVs was provided by BOKE bioscience (BOKE bioscience, Beijing, China). 500 ng libraries were pre-heated with Human cot-1(Boke bioscience) and blocker (oligo DNA by Synbio Technologies, Suzhou, China), then hybridized with HPV probes using 2 × Hybridization Buffer and Hybridization Enhancer. The hybridization mix was incubated at 95℃ for 10 min, then at 65℃ for 16 h. Dynabeads™ M-270 Streptavidin (Thermo Fisher Scientific) were used for pull down of target sequences and washing steps, then the eluted fragments containing the targeted sequences were re-amplified by 30cycles to generate sequencing library. The enriched library was quantified with the Qubit dsDNA HS Assay Kit using a Qubit 3.0 fluorometer (Thermo Fisher Scientific), and library quality was checked using Agilent 2100 TapeStation (Agilent Technologies, CA, United States). 50 ng enriched each library was pooled and attached with rapid sequencing adapters, then sequencing was performed using PromethION (FLO-PRO002) for 64 h runtime to ensure > 0.3 Gb sequence data was generated for each library.

### Data analysis

The base calling was performed using the Guppy, then the clean data was processed using Nano pack that conducted length filtering on pass reads with Q-value ≥ 7 and sequence with read-length less than 500 bp were removed. The clean reads were aligned against human genome reference (hg 19) and HPV refseq databases using a in-house bioinformatic pipeline to obtain HPV sequences and viral integration sites [17].

### Functional enrichment analysis

The reads number that detected in 12 clinical samples in this study was made into the gene expression matrix, and it was divided into two groups and named BEFORE and AFTER group. Ontology gene sets dataset of MsigDB [18, 19] was used for enrichment, the other parameters are enriched and analyzed according to the default parameter settings. The gene sets with *P* value ≤ 0.05 were regarded as the significantly enriched gene sets, and the enrichment result of the top 20 were analyzed.

## Result

### HPV abundance changes along the course of treatment

**Fig. 1:**
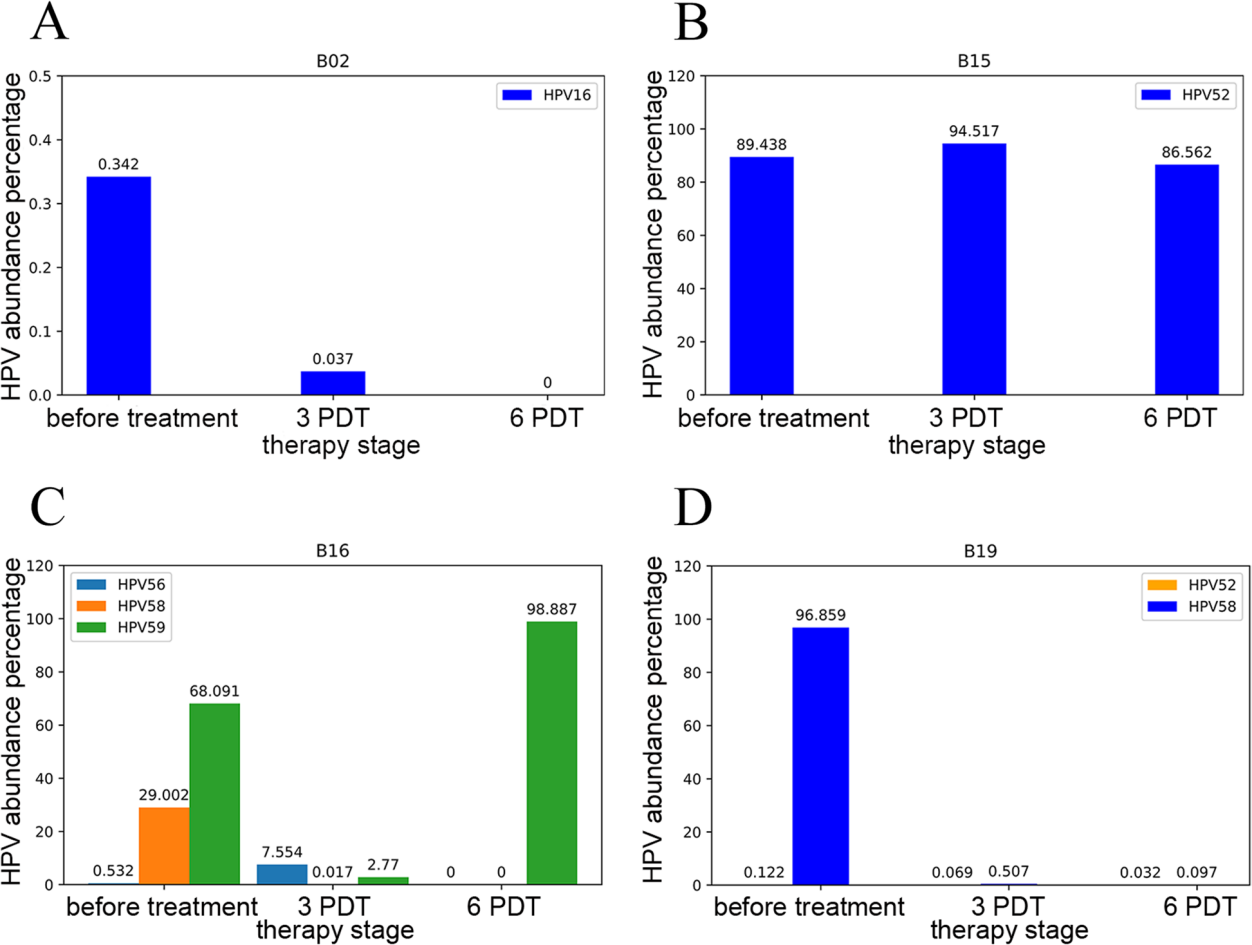
HPV abundance statistics of four patients. **A** Changes in virus abundance during three phases of treatment in patient B02 infected with HPV16. **B** Changes in virus abundance during three treatment phases in patient B15 infected with HPV52, this case has a persistent viral infection. **C** Changes in virus abundance during three treatment phases in patient B16 infected with HPV56, HPV58 and HPV59. This case was a multitype virus infection. **D** Changes in virus abundance during three treatment phases in patient B19 infected with HPV52 and HPV58. The virus was largely cleared after treatment in this case

Changes of viral abundance from thin cytology test (TCT) samples collected before treatment, after PDT 3 times, and after PDT 6 times. The abundance of HPV16 in patient B02 were observed decreasing and completely cleared after treatment (Fig. 1A). The abundance of HPV 52 in patients B15 were observed increasing after 3 PDT, but decreasing after 6 PDT, HPV virus was not cleared (Fig. 1B), but the remission of symptom was observed. The profiling of multi-infections with HPV56, HPV58 and HPV59 in patients B16 were changed along the course of treatment. The patient B16 was observed relapsed after treatment with infection of HPV59, the HPV56 and HPV58 were cleared after treatment (Fig. 1C). The abundance of HPV52 and HPV58 were hardly detected after 3 PDT in patient B19, and that may suggest a fast remission of the infection (Fig. 1D). In general, compared with qPCR results, nanopore sequencing can accurately identify the virus infection in TCT samples, even when viral load below the limit of detection (LOD) of qPCR. For instance, B15 patient was negative after 6 times of PDT by qPCR, but nanopore sequencing results showed that the virus was still detectable in the patient (Fig. 1B), and the infection was not completely cleared, so further treatment was recommended.

### Profiling changes of HPV integration sites along the course of treatment

**Fig. 2:**
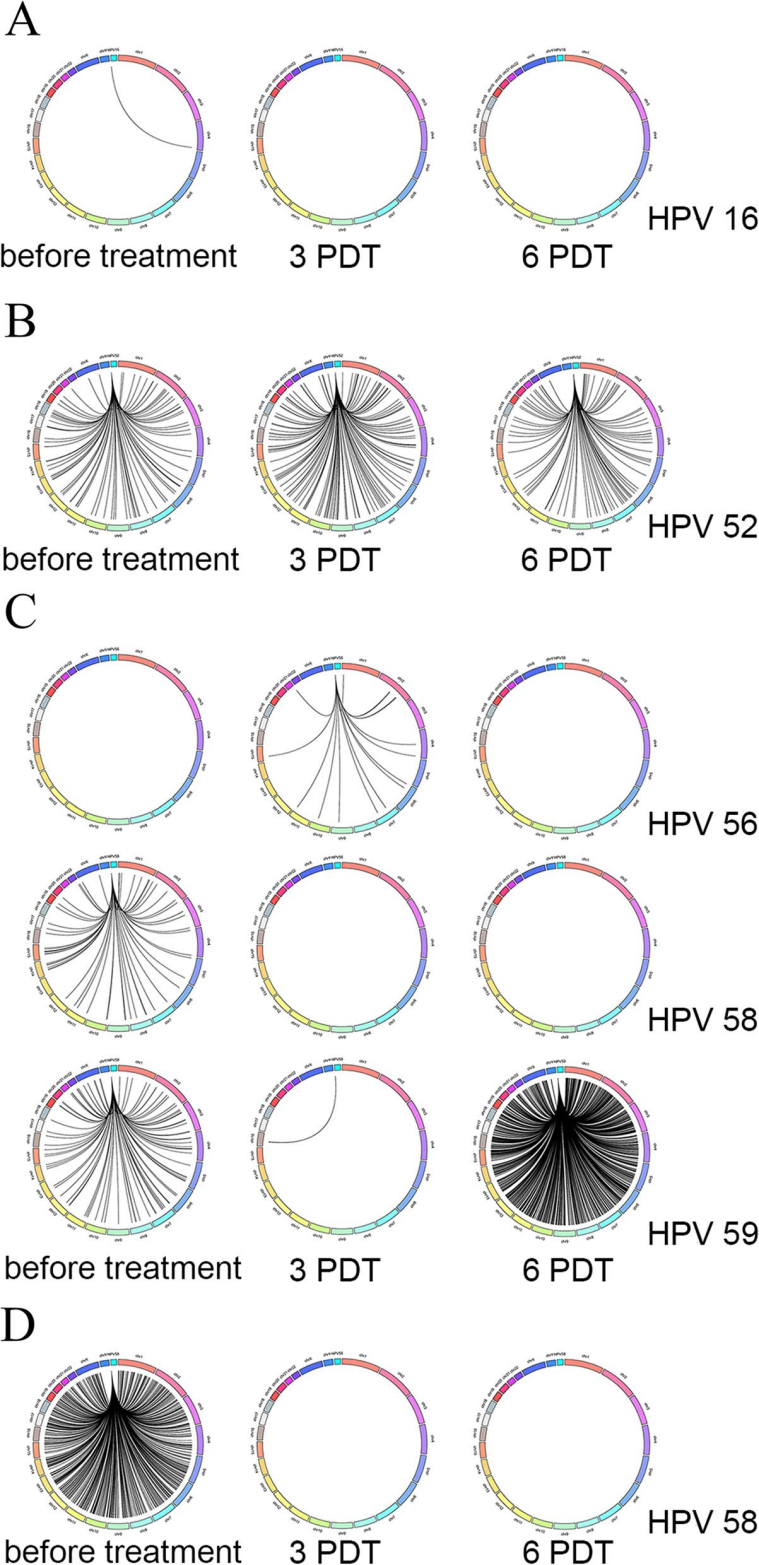
Profiling changes of HPV integration sites of four patients. **A** HPV16 integration site profiles in patient B02 during three treatment phases. **B** HPV52 integration site profiles in patient B15duiring three treatment phases. **C** HPV56, HPV58 and HPV59 integration site profiles in patient B16 during three treatment phases. **D** HPV58 integration site profiles in patient B19 during three treatment phases, HPV52 is not integrated into the human genome

The changes of HPV integration profiling in human genome of patients before and after treatment were consistent with the virus abundance. The patient B02 had very little viral integration sites before treatment (Fig. 2A), that was also consistent with pathological test results of HPV16 infection and the CIN I lesion grade, and the virus was found completely cleared after treatment. The number of HPV52 integration sites in patient B15 did not decrease after treatment (Fig. 2B), that indicated the patient had persistent infection and additional treatment was required. The patient B16 was infected with multiple HPV genotypes and each genotype had its own integration profiling. The number of integration sites of HPV56 and HPV58 was completely cleared after treatment (Fig. 2C). However, the number of integration sites of HPV59 had significantly increased after treatment, that indicated relapse of the disease and HPV integration profiling kept changing along the treatment. The number of viral integration sites in patient B19 was significant decreased after 3 PDT and completely cleared after 6 PDT (Fig. 2D).

### Persistent infection of HPV after treatment

Patient B16 was retested at 4 and 8 months after completion of treatment and the results suggested persistent positivity for HPV56 and HPV59, while the TCT results suggested low-grade squamous intraepithelial lesion** (**LSIL**)** twice (Table 2). The patient refused further nanopore sequencing and pathology testing.

**Table 2:** Patient B16 information after treatment

Sample ID	Age	Sample type	Therapy stage	HPV type(qPCR)	TCT	Postoperative pathological
B16	52	Cervical exfoliated cell	6 PDT	56、59	negative	cervicitis
B16-1	52	Cervical exfoliated cell	4 months	56、59	LSIL	N/A
B16-2	52	Cervical exfoliated cell	8 months	56、59	LSIL	N/A

### Functional annotation and enrichment analysis

**Fig. 3:**
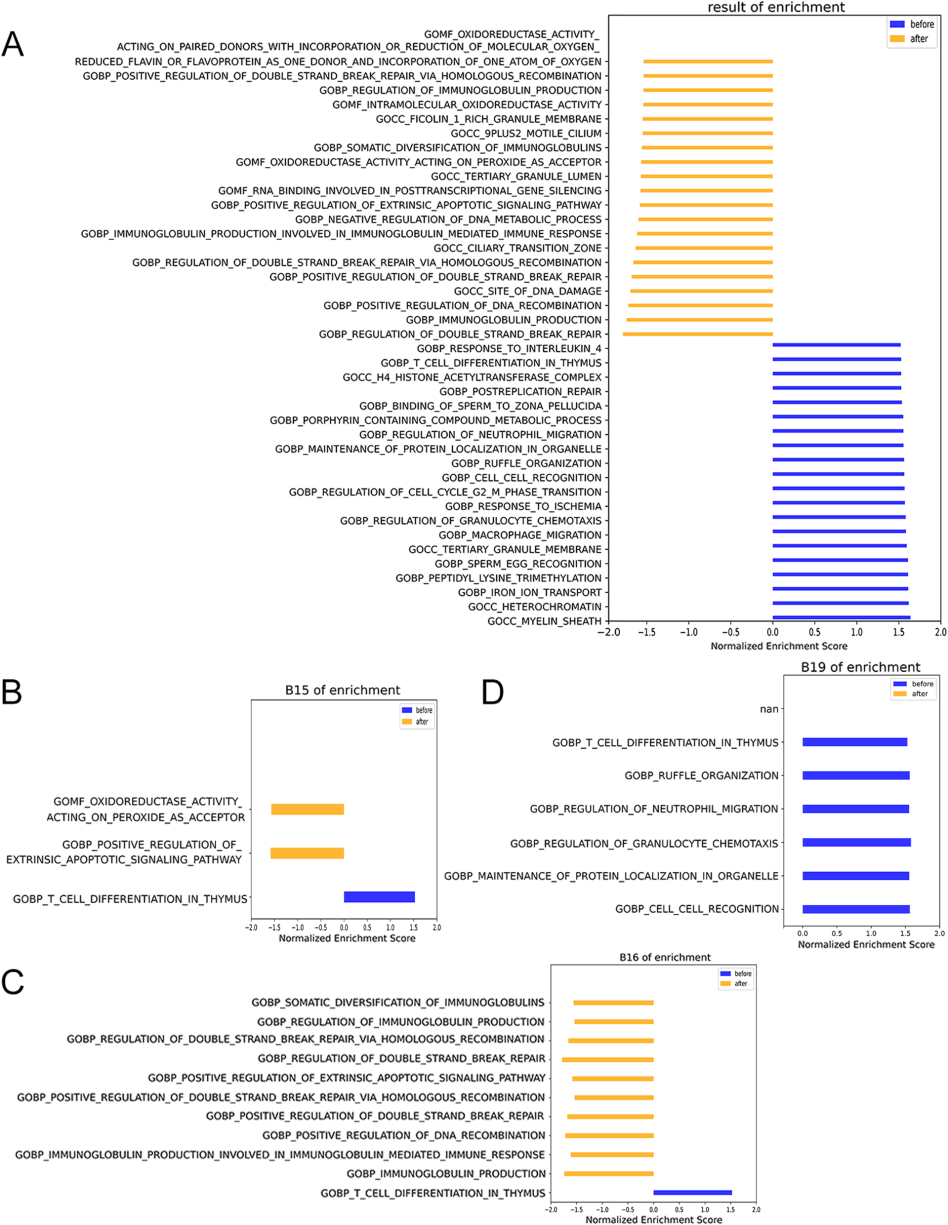
Summary of GSEA results in 3 patients. The B02 patient was not enriched in the gene sets associated with cervical cancer. **A** 40gene sets enriched before and after treatment. **B** Gene sets associated with cervical cancer development enriched in patient B15 before and after treatment. **C** Gene sets associated with cervical cancer development enriched in patient B16 before and after treatment. **D** Gene sets associated with cervical cancer development enriched in patient B19 before and after treatment

Sorting through 40 gene sets (Fig. 3A) inserted into the human genome by HPV viruses from four patients. Before treatment, 4 gene sets belonged to Cellular component and 16 gene sets belonged to Biological process. After treatment, 5 gene sets belonged to Cellular component, 11 gene sets belonged to Biological process, and 4 gene sets belonged to Molecular function, the gene sets enriched in the three ontologies of Go Term changed before and after treatment. According to the results of patient B15 (Fig. 3B), CTNNB1 related to the development of cervical cancer was found before treatment (Table 3); ATF3 and GPX3 related to cervical cancer progression were found after treatment (Table 4), that indicated the HPV integration profiling changes along the course of treatment and the patient needs further follow-up examinations. The same gene CTNNB1 was also found with HPV integration in the sample of patient B16 before treatment (Fig. 3C, Table 3), and five genes IL10, IL6, RAD51AP1, MGMT, WWOX were found after treatment (Table 3). In the sample of patient B19, four cancer related genes CCR7, AKT1, SLIT2 and CDK6 were found with HPV integrations before treatment ( Fig. 3D, Table 3), and none was found after treatment. It indicated a completed clearance of viral infection and integration at once in this patient, that was also consistent with remission of the disease and low risk of carcinogenesis after treatment.

**Table 3:** Gene information related to cervical cancer before treatment

Gene	RANK METRIC SCORE	Gene set	Patient	Possible effect of cervical cancer
CTNNB1	0.146	GOBP_T_CELL_DIFFERENTIATION_IN_THYMUS	B15, B16	promote tumorigenesis
CCR7	0.357	GOBP_T_CELL_DIFFERENTIATION_IN_THYMUS	B19	influence of lymph node metastasis in cervical cancer
		GOBP_RUFFLE_ORGANIZATION		
		GOBP_REGULATION_OF_NEUTROPHIL_MIGRATION		
		GOBP_REGULATION_OF_GRANULOCYTE_CHEMOTAXIS		
		GOBP_CELL_CELL_RECOGNITION		
CDK6	0.357	GOBP_T_CELL_DIFFERENTIATION_IN_THYMUS		affect the development of cervical precancerous lesions
SLIT2	0.357	GOBP_REGULATION_OF_NEUTROPHIL_MIGRATION		potential predictive biomarkers for cervical cancer patients receiving chemotherapy or radiotherapy postoperatively
		GOBP_REGULATION_OF_GRANULOCYTE_CHEMOTAXIS		
AKT1	0.417	GOBP_MAINTENANCE_OF_PROTEIN_LOCALIZATION_IN_ORGANELLE		malignant progression of cervical cancer

**Table 4:** Gene information related to cervical cancer after treatment

Gene	RANK METRIC SCORE	Gene set	Patient	Possible effect of cervical cancer
GPX3	-0.226	GOME_OXIDOREDUCTASE_ACTIVITY_ACTING_ON_PEROXIDE_AS_ACCEPTOR	B15	biomarker of lymph node metastasis and prognosis in cervical cancer
ATF3	-0.226	GOBP_POSITIVE_REGULATION_OF_EXTRINSIC_APOPTOTIC_SIGNALING_PATHWAY		tumor-inhibiting factor
IL6	-0.226	GOBP_IMMUNOGLOBULIN_PRODUCTION	B16 B16	cervical cancer progression and metastasis
		GOBP_REGULATION_OF_IMMUNOGLOBULIN_PRODUCTION		
IL10	-0.226	GOBP_IMMUNOGLOBULIN_PRODUCTION		references for the diagnosis of cervical cancer
		GOBP_IMMUNOGLOBULIN_PRODUCTION_INVOLVED_IN_IMMUNOGLOBULIN_MEDIATED_IMMUNE_RESPONSE		
		GOBP_REGULATION_IMMUNOGLOBUL_PRODUCTION		
		GOBP_SOMATIC_DIVERSIFICATION_OF_IMMUNOGLOBULINS		
PAD51AP1	-0.226	GOBP_POSITIVE_REGULATION_OF_DNA_RECOMBINATION		increase the susceptibility of cancer cells to radiotherapy
		GOBP_POSITIVE_REGULATION_OF_DOUBLE_STRAND_BREAK_REPAIR		
		GOBP_POSITIVE_REGULATION_OF_DOUBLE_STRAND_BREAK_REPAIR_VIA_HOMOLOGOUS_RECOMBINATION		
		GOBP_REGULATION_OF_DOUBLE_STRAND_BREAK_REPAIR		
		GOBP_REGULATION_OF_DOUBLE_STRAND_BREAK_REPAIR_VIA_HOMOLOGOUS_RECOMBINATION		
MGMT	-0.226	GOBP_POSITIVE_REGULATION_OF_DOUBLE_STRAND_BREAK_REPAIR		promotes the anti-proliferative
WWOX	-0.226	GOBP_POSITIVE_REGULATION_OF_EXTRINSIC_APOPTOTIC_SIGNALING_PATHWAY		associated with cervical cancer development

## Discussion

Based on the analysis of HPV abundance, profiling changes of HPV integration sites and the gene set related to cervical cancer progression after enrichment, we developed a nanopore sequencing based assay as companion diagnostic tools to conduct individualized treatment. As HPV abundances increased, HPV integration sites were found increasing correspondingly, and more genes related to cervical cancer were also observed. The advantage of this assay over traditional qPCR tests, was not only better sensitivity (we can identify enriched viral reads when viral load below the LOD of qPCR) to avoid misdiagnosis, but also quantitatively detection of viral abundance and integration sites at once, moreover, with enriched reads over viral genome, we could more accurately identify viral genotype, sub-genotypes or even drug-resistant variations.

The HPV genome is divided into three regions: (i) the long control region (LCR) which contains binding sites of viral regulatory proteins (E1 and E2) and cellular transcription factors, (ii) the early region (E) that encodes for the E1, E2, E4, E5, E6 and E7 genes and (iii) the late region (L) that encodes for the L1 and L2 genes [6, 20, 21]. Over the last few decades, the strong tumourigenic capacity of HPV16 DNA triggered the extensive sequence analysis of the viral genome, several nucleotide changes have been associated with immune escape, viral persistence and cervical cancer development [22, 23]. HPV16 is a strong carcinogenic factor, because it is detected in more than 50% of cervical cancer cases. The tumourigenic hallmark of HPV16 DNA lies mainly on the function of E6 and E7 oncoproteins and numerous analyses have proved that mutations particularly within E6 increase the oncogenicity of viral DNA [24–26]. The T350G (L83V) was a nucleotide variation of HPV16 DNA on the E6 gene, implicated in the development of severe dysplasia in various geographic populations. In vitro experiments demonstrated that the coexistence of nucleotide changes G145T (Q14H), C335T (H78Y) along with T350G (L83V) considerably augment the tumourigenic capacity of the viral oncoprotein [27, 28]. To date, no nucleotide variations have been involved in the development of cervical disease concerning E4 gene. Conversely, the nucleotide change A4042G/T (I65V) in the E5 oncogene seems to be mainly identified in specimens diagnosed with advanced cervical dysplasia [29–31]. The LCR is regarded as the most polymorphic region of viral DNA, with numerous novel nucleotide variations to be continually reported in diverse research studies.

HPV infects the host cells and usually exists in a free state, HPV latent in the basal cell layer, where its DNA moves into the nucleus of infected cells in an independent exogenous chromosomal state. Subsequently, HPV integrates into the host chromosome, leading to genomic instability and tumorigenesis of the host. Upon integration the circular episomal HPV DNA is converted into a linear structure and inserted into the host chromosome. The disruption of viral genome can take place within the early region of viral genome (E1 and E2 genes) [32–34], or within the late region (L1 and L2 genes) [35, 36]. The most frequent site of disruption within the E2 ORF is located in the portion that encompasses the hinge region of the E2 protein [34, 35, 37]. In contrast, disruptions within E1 gene have been found to be more frequently located either at the 5′ end [32, 38], or at the 3′ end of the E1 gene [36].

HPV is often present in cervical lesion cells with a mixed infection state, for instance, 2 different genotypes of HPV were found in patient B19, with HPV58 in the integrated state and HPV52 in a free state. Integration of HPV into the host chromosome is a key factor in cervical carcinogenesis. Genes related to cervical cancer progression was found by enrichment analysis before treatment, including CTNNB1, CCR7, AKT1, SLIT2, CDK6, etc. Human SAIL4 (sal-like 4), the new discovered proto oncogenes encode transcription factors that acted at the level of stem cells, SAIL4 protein can recognize and bind to CTNNB1 promoter region and trans‐activating CTNNB1 for accelerated expression β- Catenin, which promoted cell proliferation and the formation of tumor cervical cancer cells [39]. Invasion and metastasis of tumor cells through the bloodstream and lymphatic vessels were key steps in the progression of cervical cancer, CCR7 was implicated in mediating lymphocyte trafficking and spreading to lymph nodes, expression of CCR7, CXCR4, VEGF-C and VEGF-D may have a synergistic effect on the malignant development of cervical cancer and lymph node metastasis [40]. Akt1 and p-Akt1 were key elements of PI3K/Akt signaling pathways that regulate cellular processes, including proliferation, differentiation, migration and survival, the HK2 (the member of hexokinases, Associated with malignant tumor growth and distant metastasis) and AKT1 (p-AKT1) may regulated in the same networks in cervical cancer cells and synergistically promoted malignant growth and distant metastasis during the development of cervical cancer [41]. It had been shown that SLIT2 expression levels were negatively correlated with the incidence of cervical cancer in patients treated with postoperative chemotherapy (CT) or radiotherapy (RT), indicating that SLIT2 may be a potential predictive biomarker [42]. Cyclin-dependent kinase6 (CDK6) was a crucial regulatory cancer-related gene within the cell cycle and tumorigenesis, the changes in the level of CDK6 expression may impact on the progression of cervical precancerous lesions in the female [43]. HPV integration frequency increases with severity of cervical precancer, and viral integrations are present in the majority of cervical cancers. In this study, patients B02 and B16, both had a pathological grade of CIN 1, and had a lower pathological grade than patients B15 and B19 with CIN 2, patients B02 and B16 had lower viral abundance and significantly fewer integration sites than the latter.

The principle of photodynamic therapy is to selectively kill actively proliferating cells. The more severe of cervical lesion, the more actively proliferating of its cells, with a greater number of HPV insertion sites and a higher HPV abundance. After photodynamic therapy, the actively proliferating cervical cells are killed and the degree of cervical lesions is significantly reduced. The integrated HPV viruses were cleared, so the HPV abundance and the number of integration sites decreased significantly in patients B02 and B19. However, in patients B15 and B16, we found that although their cervical pathological grade decreased after photodynamic therapy, their HPV abundance and the number of integration sites did not decrease accordingly, considering that the persistent HPV infection lead to new HPV integration. Especially for patient B16, the HPV59 abundance and the number of integration sites increased significantly after 3 PDT, the possible reason for this being the ineffectiveness of PDT and predicting the persistent infection of HPV and a possible exacerbation of the cervical lesions. Subsequent follow-up results also confirmed the presence of persistent infection of HPV56 and HPV59, while the cervical lesions developed from cervicitis to LSIL. Its possible explanation was the production of new HPV integration. Nanopore technology can predict the direction of disease progression earlier than TCT and pathology. Regrettably, patient B16 refused any further HPV integration site testing and pathology testing. We can still identify some genes related to cervical cancer progression from the samples of the two patients after treatment, including ATF3, GPX3, IL10, IL6, RAD51AP1, MGMT, WWOX. Activated transcription factor 3 (ATF3) was a stress-induced transcription repressor, it had been shown that ATF3 overexpression enhanced paclitaxel apoptosis in cervical cancer HeLa cells in part by stimulating TAp73, thus, it can act as a tumor suppress factor [44]. Glutathione peroxidase 3 (GPX3) was the member of the glutathione peroxidase family of selenoproteins, GPX3 promoter methylation was one of the main causes of downregulated GPX gene expression in cervical cancer and acted as a predictive biomarker for lymph node metastasis and prognosis in cervical cancer [45]. In cervical cancer tissues, the expression levels of IL10 and Ki-67 increased accordingly when the HPV infection rate increased, so the detection of IL10, KI67 expression and HPV infection rate can provide reference for the diagnosis of cervical cancer [46]. Thrombocythemia, high PLR (platelet to lymphocyte ratio) and IL-6 overexpression correlated with cervical cancer progression and metastasis, they may play an important role as molecular markers of survival and may be considered as potential predictors of cervical cancer prognosis [47]. RAD51AP1 was the main BRD4 target gene participating in radiosensitivity, and high expression of BRD4 was found to be associated with poorer prognosis and radioresistance. JQ1 (a BRD4 inhibitor) sensitized cervical cancer to radiation therapy by suppressing RAD51AP1 transcription [48]. Studies had shown that 5-Aza-dC (DNA methyltransferase inhibitor) and X-ray radiation can inhibit expression of p16 (cyclin-dependent kinase inhibitor 2A, CDKN2A) and MGMT in cervical cancer cells, and down regulating the expression of p16 and MGMT can promote the anti-proliferation and treatment of cervical cancer cells, this discovery may provide a new treatment and a new method for cervical cancer [49]. It had been shown that WWOX is under-expressed in cervical cancer tissues and cell lines, and the expression of WWOX significantly decreases or disappears with the development of cervical cancer, proved that the reduced expression of WWOX is strongly associated with the development of cervical cancer, regulating the expression of WWOX may be an effective and novel method to treat cervical cancer [50]. Therefore, these two patients are still at high risk of relapse, even the clinical remission were observed. The prognosis of cervical lesions cannot be determined by pathological diagnosis alone, but also by integrating information on HPV abundance and integration profiling changes, especially those HPV integrated genes/networks related to carcinogenesis.

In this study, we developed a nanopore-sequencing based assay that provides information on HPV infection, genotype and integration all at once for patients with cervical lesions along the course of photodynamic therapy. The technique advantages of viral sequence enrichment combined with nanopore long-read sequencing contributed to more accurate prognosis determination as well as the dynamic monitoring of treatment accompaniment for a better evaluation of photodynamic therapy. That makes it a promising companion diagnostic tools and favorable supplement to traditional pathological examinations.

## Data Availability

The datasets generated and/or analysed during the current study are available in the SRA repository, BioProject No.: PRJNA942458.

## Abbreviations

WHO: World Health Organization
HPV: Human papillomavirus
PCR: Polymerase chain reaction
ALA-PDT: Aminolevulinic acid photodynamic therapy
CIN: Cervical intraepithelial neoplasia
PDT: Photodynamic therapy
TCT: Thin cytology test
LOD: Limit of detection
qPCR: Quantitative polymerase chain reaction
LSIL: Low-grade squamous intraepithelial lesion
CTNNB1: Catenin (cadherin-associated protein), beta 1
ATF3: Activated transcription factor 3
GPX3: Glutathione peroxidase 3
IL10: Interleukin 10
IL6: Interleukin 6
RAD51AP1: RAD51 associated protein 1
MGMT: O-6- methylguanine-DNA methyltransferase
WWOX: WW domain containing oxidoreductase
CCR7: Chemokine (C–C motif) receptor 7
SLIT2: Slit homolog 2
CDK6: Cyclin-dependent kinase6
SAIL4: Sal-like protein 4
CXCR4: Chemokine (C-X-C motif) receptor 4
VEGF-C: Vascular endothelial growth factor C
VEGF-D: Vascular endothelial growth factor D
PI3K/Akt: Phosphatidylinositide 3-kinases/ protein kinase B
HK2: Hexokinase 2
PLR: Platelet to lymphocyte ratio
BRD4: Bromodomain containing 4
5-Aza-dC: 5-Aza-2'-deoxycytidine
CDKN2A: Cyclin-dependent kinase inhibitor 2A
CT: Chemotherapy
RT: Radiotherapy
